# Psychosocial work factors and social inequalities in psychological distress: a population-based study

**DOI:** 10.1186/s12889-017-4014-4

**Published:** 2017-01-18

**Authors:** Caroline S. Duchaine, Ruth Ndjaboué, Manon Levesque, Michel Vézina, Xavier Trudel, Mahée Gilbert-Ouimet, Clermont E. Dionne, Benoît Mâsse, Neil Pearce, Chantal Brisson

**Affiliations:** 1 CHU de Québec-Université Laval Research Center, Population Health and Optimal Health Practices Unit, Saint-Sacrement Hospital, 1050 chemin Sainte-Foy, Quebec city, G1S 4L8 QC Canada; 2 Social and preventive medicine department, Faculty of Medicine, Laval University, 1050 avenue de la Médecine, Quebec city, G1V 0A6 QC Canada; 3 Rehabilitation department, Faculty of Medicine, Laval University, 1050 avenue de la médecine, Quebec city, G1V 0A6 QC Canada; 4 Social and preventive medicine department, Public Health School, Montreal University, 7101 avenue du Parc, Montreal, H3N 1X9 QC Canada; 5 Faculty of Epidemiology and Population Health, London School of Hygiene and Tropical Medicine, Keppel Street, WC1E 7HT London, UK; 6 Research Center CHU-Ste-Justine, 3175 Côte Ste-Catherine, Montréal, H3T 1C5 Québec Canada

**Keywords:** Social inequalities, Mental health problems, Job strain, Effort-reward imbalance, Psychological distress

## Abstract

**Background:**

Mental health problems (MHP) are the leading cause of disability worldwide. The inverse association between socioeconomic position (SEP) and MHP has been well documented. There is prospective evidence that factors from the work environment, including adverse psychosocial work factors, could contribute to the development of MHP including psychological distress. However, the contribution of psychosocial work factors to social inequalities in MHP remains unclear. This study evaluates the contribution of psychosocial work factors from two highly supported models, the Demand-Control-Support (DCS) and the Effort-Reward Imbalance (ERI) models to SEP inequalities of psychological distress in men and women from a population-based sample of Quebec workers.

**Methods:**

Data were collected during a survey on working conditions, health and safety at work. SEP was evaluated using education, occupation and household income. Psychosocial work factors and psychological distress were assessed using validated instruments. Mean differences (MD) in the score of psychological distress were estimated separately for men and women.

**Results:**

Low education level and low household income were associated with psychological distress among men (MD, 0.56 (95% CI 0.06; 1.05) and 1.26 (95% CI 0.79; 1.73) respectively). In men, the contribution of psychosocial work factors from the DCS and the ERI models to the association between household income and psychological distress ranged from 9% to 24%. No clear inequalities were observed among women.

**Conclusions:**

These results suggest that psychosocial work factors from the DCS and the ERI models contribute to explain a part of social inequalities in psychological distress among men. Psychosocial factors at work are frequent and modifiable. The present study supports the relevance of targeting these factors for the primary prevention of MHP and for health policies aiming to reduce social inequalities in mental health.

**Electronic supplementary material:**

The online version of this article (doi:10.1186/s12889-017-4014-4) contains supplementary material, which is available to authorized users.

## Background

Evidence is accumulating on social inequalities in health. [1–3]. These social inequalities are characterized by higher risk of poor physical and mental health among people in more disadvantaged socioeconomic position (SEP). Social inequalities in mental health problems (MHP) have previously been documented in several studies [4, 5]. MHP are the leading cause of disability worldwide [6]. Their prevalence, long duration and high risk of recurrence [7] place a considerable burden on health and social care systems and important productivity losses for employers [6]. Understanding the pathways that link SEP to MHP is therefore of important public health significance [3].

Work environment has been suggested to act as one such pathway [8, 9]. Factors from the work environment, including adverse psychosocial work factors, were shown to contribute to the development of MHP, including psychological distress [10–13]. Evidences also suggest that low SEP workers tend to concentrate in jobs where the prevalence of exposure to adverse psychosocial work factors is high [14, 15].

Two theoretical models have been widely used to measure the effect of psychosocial work factors on health, the Demand-Control-Support (DCS) [16] and the Effort-Reward Imbalance (ERI) models [17]. The DCS model states that workers simultaneously exposed to high psychological demands and low job control, i.e. *job strain*, are more at risk to develop health problems. A third component, low social support from colleagues and supervisor may act directly or amplify the effect of job strain [18]. The ERI model proposes that workers are in a state of detrimental imbalance when high efforts are accompanied by low reward (respect, esteem, and promotion prospect), and thus more susceptible to health problems [17, 19]. The proportions of working men and women exposed to these adverse factors have been found to be about 20–25% in previous prospective studies conducted in industrialized countries [20]. These factors were also shown to be modifiable through workplace interventions [21, 22].

Previous studies that have examined the contribution of psychosocial work factors to social inequalities in MHP showed inconsistent results [15, 23–36]. Three previous studies have examined the contribution of ERI exposure at work [23, 34, 36], which deleterious effect on mental health is well-documented and showed to be independent from other adverse psychosocial factors at work [37–40]. Only one previous study has examined the relative and complementary contribution of DCS and ERI exposures in explaining social inequalities in mental health, using four dimensions covered by these theoretical models [36].

The primary aim of this study was to evaluate the contribution of psychosocial work factors from the DCS and the ERI models in the SEP inequalities of psychological distress. We also examined the additional contribution of other psychosocial work-related factors and other works-related factors in these inequalities. We hypothesized that psychosocial work factors from the DCS and the ERI models are important contributors to social inequalities of psychological distress. The contribution was evaluated for three SEP indicators – education, occupation and household income, and for men and women separately.

## Methods

### Study population and recruitment procedure

Data used in this cross-sectional analysis were collected as part of the Quebec survey on working conditions, employment, health and safety at work (EQCOTESST). The population and recruitment procedures have been described in details elsewhere [41]. Briefly, the study population consists of all Quebec workers aged 15 years-old or more, who were employed for at least eight weeks and worked for at least 15 h per week. Workers who lived in institutions, on Indian reserves or military workers were excluded. Sampling method of EQCOTESST was done in two steps. Firstly, a random digit dialing sampling was made among people with a fixed telephone line to select eligible household. Secondly, one participant per household was randomly selected among eligible workers within household. In order to ensure that the sample represented all Quebec workers, recruitment was made by strata proportionally to Quebec’s administrative regions. A total of 5071 workers (2632 men and 2439 women) participated in the survey, with a participation rate of 62%. For the purpose of the present study, self-workers were excluded from the analysis (*n* = 659) because of an elevated number of missing data on psychosocial work factors variables. Thus, the final study sample was composed of 4412 employees (2270 men and 2142 women).

### Data collection

Data were collected between November 2007 and February 2008 by telephone interviews. Participants to the EQCOTESST survey were shown to have similar sociodemographic characteristics to those of participants of other major Canadian surveys, namely “L’Enquête sur la population active” (EPA) and “L’Enquête sur la santé dans les collectivités canadiennes” (ESCC) [41].

#### Socioeconomic position

SEP was defined with three indicators, education (less than high school degree, high school degree, college degree, and university degree), occupation (unskilled workers and maneuvers, qualified workers, office workers, overseers and first level managers, semi-professionals and technicians, professionals, senior and middle managers) and household income (0–39 999, 40 000–59 999, 60 000–99 999 and ≥100 000 CAN$/year). Occupation was initially classified according to the National Occupation Classification of Canada (4-digit code, 2006) and then grouped into four categories based on the hierarchical level, formation level and type of work [41].

#### Psychosocial work factors from the DCS and the ERI models

The questions used to measure each component of the DCS and the ERI models originate from validated versions and their internal consistencies were measured in a representative sample of Quebec’s working population, **Psychological demands** (PD) were evaluated with five items from the 6-item, short French version of the Job Content Questionnaire (JCQ) [42] and one item from the 9-item version of the JCQ (Cronbach’s α = 0.72) [41]. **Job control** (JC) were measured using five items adapted from the JCQ (Cronbach’s α = 0.61) [41]. **Social support at work** (SS) was evaluated with six items from the French version of JCQ and one question from the Copenhagen questionnaire on psychosocial factors at work (COPSOQ), “*At my work, I have the impression to be part of a team*” [43]. The internal consistency of this scale for this sample was shown to be good (Cronbach’s α = 0.83) [41]. PD, JC and SS were categorized in tertiles. Regarding the ERI model, **reward** at work was measured using six items from the ERI validated scale [44] with the addition of two other validated items from the COPSOQ [43]. Internal consistency of this scale was also shown to be good (Cronbach’s α = 0.81) [41]. The score of reward at work was categorized in tertiles and the first tertile (lowest reward) was considered as the most exposed group.

#### Other psychosocial work-related factors

A set of seven self-reported items was used to measure other psychosocial work-related factors. 1- **Job contractual instability** was considered present when one of the following situations appeared: the subject was working part-time (15 to 29 h per week) and wanted more work hours, had obtained his job by an agency or had a fixed term employment. 2- **Psychological harassment** was evaluated with one question which was based on the legal definition of the Quebec law on labor standard “*During the past 12 months at your current main job*, *were you subjected to psychological harassment*, *that is*, *repeated verbal harassment or actions that affected your dignity or personal integrity?*” 3- The availability of **flexible work schedule** and 4-**paid leaves for sickness** in the workplace were both evaluated with one question (present or not). 5- **Emotionally demanding work**, 6- **strain with public** and 7- **possibility to do a work of quality** were also evaluated with one self-reported question each. These three last items measured demanding work situations.

#### Other work-related factors

A set of five items was used to measure other work-related factors, 1- **work schedule** (working on day, evening or night shift; and regular, rotating or other schedule), 2- **number of working hours** (<30, 30–39, 40, or >40 h per week), 3- **self-reported exposure to noise** (“*In your main employment*, *how often are you working in an environment where it is so noisy that it is difficult to hold a conversation with someone a few feet or one meter from you*, *even when shouting?*” [45]), 4- **self**-**reported exposure to solvents** (“*In your main employment*, *how often are you inhaling vapors of solvents such as paint strippers*, *oil paint*, *thinners*, *varnish*, *Varsol*, *turpentine*, *etc*.” [46]), and 5- **physical constraints**. This last work factor was evaluated with nine items regarding movement, posture, physical effort and vibration exposure that are considered risk factors to musculoskeletal problems [41]. (See Additional file 1 for more detailed information about work-related factors).

#### Psychological distress

Psychological distress was measured with the K6, a 6-item instrument designed and validated by Kessler et al. [47]. Psychological distress measured with the K6 has been consistently shown to predict mental disorders [48]. Each item was based on a four-point Likert scale and the sum of the six items was calculated to obtain the score of psychological distress. This score varied from 0 (no distress) to 24 (maximum distress) [47, 49].

### Statistical analysis

All analyses were weighted in order to infer conclusions to the target population. First, the inverse of the probability of being selected was calculated. Second, adjustment was made for non-response observed in household and non-response observed in the selected sample of workers. Finally, the weights were corrected for the underrepresentation of private households with no fixed telephone line.

Mean differences (MD) in the score of psychological distress were modeled using ANCOVA. MD were calculated for psychological distress using each SEP indicator. Work-related factors were included in age-adjusted models in five steps. 1- Psychosocial work factors of DCS and ERI models (PD, JC, reward and SS in tertiles) were introduced one by one to measure the contribution of each component separately. 2- The psychosocial work factors from the DCS and the ERI models which present a positive contribution to the SEP inequalities were then introduced together in a model. This last model was retained for further sequential adjustments. 3- The set of the seven other psychosocial work-related factors was then added to the previous model. 4- The set of the five other work-related factors was finally added to obtain a fully-adjusted model. Each analysis was conducted separately for men and women. The contribution of each component and/or set of factors was measured with the MD obtained from the ANCOVA using the following formula [50],

$$ \left[\left({\mathrm{MD}}_{\mathrm{basic}}\hbox{--}\ {\mathrm{MD}}_{\mathrm{adjusted}}\right)/\left({\mathrm{MD}}_{\mathrm{basic}}\right)\right]*100 $$

$$ {\mathrm{MD}}_{\mathrm{basic}} = \mathrm{age}\hbox{-} \mathrm{adjusted}\ \mathrm{M}\mathrm{D} $$

$$ {\mathrm{MD}}_{\mathrm{adjusted}} = \mathrm{M}\mathrm{D}\ \mathrm{adjusted}\ \mathrm{f}\mathrm{o}\mathrm{r}\ \mathrm{age}\ \mathrm{and}\ \mathrm{f}\mathrm{o}\mathrm{r}\ \mathrm{o}\mathrm{ther}\ \mathrm{work}\hbox{-} \mathrm{related}\ \mathrm{variables} $$

This contribution was calculated for the lowest category of SEP. The Jackknife method was used to estimate 95% confidence intervals around these contributions. All analyses were performed using SAS software 9.4.

## Results

### Characteristics of the study population

The characteristics of the study population are shown in Table 1. Most of the population was aged between 25 and 44 years (48.5% of men and 45.1% of women), had at least a high school degree (84.7% of men and 90.4% of women) and were unskilled workers and maneuvers (31.5% of men and 24.6% of women). The lower quartile of household income in this population (0–39 999 $/year), which was considered the most exposed group, fell between the low income threshold for a three members family (36 889$/year) and a four members family (42 596$/year), as reported by the Quebec government in 2008 [51]. The mean score of psychological distress was higher among women (4.39) than men (3.41). Women were slightly more exposed to adverse psychosocial work factors from the DCS and ERI models (PD, JC, and reward), except for SS. Missing value were 2% or less in all variable except for household income. However, individuals with missing values on household income were comparable from individuals without missing values in terms of psychological distress and psychosocial works factors exposure (not shown).

**Table 1:** Weighted^a^ characteristics of the study population

	Men (*n* = 2270)	Women (*n* = 2142)
Age, *n* (%)		
15–24	259 (11.4)	314 (14.7)
25–44	1100 (48.5)	965 (45.1)
45–54	623 (27.5)	633 (29.6)
≥55	288 (12.7)	230 (10.7)
Education, *n* (%)		
Less than high school degree	345 (15.3)	205 (9.6)
High school degree	765 (33.9)	698 (32.8)
College degree	562 (24.9)	564 (26.5)
University degree	583 (25.9)	663 (31.1)
Occupation, *n* (%)		
Unskilled workers and maneuvers	715 (31.5)	527 (24.6)
Qualified workers	386 (17.0)	162 (7.6)
Office workers	145 (6.4)	491 (23.0)
Overseers and first level managers	320 (14.1)	409 (19.1)
Semi-professionals and technicians	171 (7.6)	88 (4.1)
Professionals	352 (15.5)	356 (16.7)
Senior and middle managers	179 (7.9)	105 (4.9)
Household income (quartiles), *n* (%)^b^		
0–39 999$	457 (21.2)	484 (25.0)
40 000–59 999$	479 (22.3)	422 (21.7)
60 000–99 999$	746 (34.7)	620 (31.9)
≥100 000$	470 (21.9)	414 (21.4)
Psychological distress, mean (SD)	3.41 (3.18)	4.39 (3.54)
Psychological demand (tertiles), *n* (%)		
0–7.2	761 (33.7)	704 (33.1)
7.3–10	938 (41.5)	778 (36.5)
>10	562 (24.8)	649 (30.4)
Job control (tertiles), *n* (%)		
0–20.9	619 (27.4)	740 (34.7)
21–24.9	753 (33.2)	662 (31.1)
≥25	892 (39.4)	728 (34.2)
Reward at work (tertiles), *n* (%)		
0–13.9	582 (26.2)	640 (30.6)
14–15.9	619 (27.8)	523 (25.0)
≥16	1025 (46.0)	932 (44.5)
Social support at work (tertiles), *n* (%)		
0–47	663 (29.5)	543 (25.6)
48–55	814 (36.2)	710 (33.6)
≥56	688 (30.6)	787 (37.2)
Working alone	85 (3.8)	78 (3.7)

^a^The sum of the frequencies can be different from the expected number because weighted data were rounded

^b^Missing values were ≤ 2% for all variables except for household income (*n* = 321 (119 men and 202 women) where 7.3% were missing)

### Social inequalities in psychological distress

Table 2 shows the age-adjusted MD for psychological distress according to three SEP indicators in men and women. Household income showed the strongest association with psychological distress among men. Men in the lowest income categories (less than 40 000$/year and 40 000–59 999$/year) present a higher score of psychological distress, compared to men in the highest income category (MD, 1.26 (95% CI 0.79; 1.73) *P* < 0.001 and 0.62 (95% CI 0.16; 1.07) *P* < 0.01, respectively). Psychological distress was also higher among men with less than a high school degree, compared to men with a university degree (MD, 0.56 (0.06; 1.05) *P* < 0.05). Furthermore, psychological distress was higher in the lowest occupation category among men and in the lowest education degree among women (MD ranging from −0.14 to 0.47, and from 0.01 to 0.32 respectively). However, these associations were not statistically significant. No clear inequalities were observed with occupation and household income among women.

**Table 2:** Mean differences of psychological distress according to three socioeconomic position indicators

	Men	Women
Psychological distress, MD (95% IC)^a^		
Education degree		
University	REF	REF
College	0.00 (−0.42; 0.43)	0.01 (−0.44; 0.47)
High school degree	0.21 (−0.18; 0.61)	0.27 (−0.17; 0.71)
Less than high school degree	0.56 (0.06; 1.05)*	0.32 (−0.33; 0.97)
*P* interaction for gender^b^ = 0.991		
Occupation		
Senior and middle managers	REF	REF
Professionals	−0.14 (−0.78; 0.51)	1.15 (0.27; 2.02)*
Semi-professionals and technicians	0.32 (−0.44; 1.07)	0.46 (−0.68; 1.60)
Overseers and first level managers	0.08 (−0.58; 0.74)	1.01 (0.27; 2.02)*
Office workers	0.32 (−0.47; 1.11)	0.69 (−0.15; 1.54)
Qualified workers	0.11 (−0.54; 0.75)	0.48 (−0.15; 1.48)
Unskilled workers and maneuvers	0.47 (−0.13; 1.07)	1.08 (0.22; 1.93)*
*P* interaction for gender^b^ = 0.1968		
Household income (quartiles) ($/year)		
≥100 000	REF	REF
60 000–99 999	0.32 (−0.10; 0.74)	0.23 (−0.27; 0.72)
40 000–59 999	0.62 (0.16; 1.07)**	0.51 (−0.03; 1.05)
0–39 999	1.26 (0.79; 1.73)***	0.43 (−0.09; 0.95)
*P* interaction for gender^b^ = 0.106		

*p-value <0.05, **p-value <0.01, ***p-value <0.001

^a^Adjusted for age

^b^ *P* for multiplicative interaction term added in the models

### Contribution of work-related factors to social inequalities in psychological distress

As shown in Table 2, the strongest social inequalities in psychological distress were observed among men, using household income as the SEP indicator. This particular case was retained to presents the contribution of work-related factors. Contributions were also calculated using education and yielded similar estimates (Additional file 2).

#### Psychosocial work factors from the DCS and the ERI models

Table 3 presents the contribution of psychosocial work factors from the DCS and the ERI models to the association between household income and psychological distress among men. Overall, the association between the lowest household income category and psychological distress was slightly attenuated after adjustment for psychosocial work factors (MD ranged from 1.26 to 0.96, Models I to VI). Among components of the DCS and ERI models, the highest contribution to the association between household income and psychological distress was found for reward (24%). JC and SS also explained part of this association with contributions of 9% and 14% respectively. In contrast, PD showed a negative contribution (−24%). Altogether, JC, reward and SS contribute to explain 23% (95% CI 5; 40) of the association (Model VI).

**Table 3:** Contribution of the Demand-Control-Support and the Effort-Reward Imbalance models to income inequalities in psychological distress among men

	Model I	Model II	Model III	Model IV	Model V	Model VI
	Age-adjusted	Model I + Psychological demand	Model I + Job control	Model I + Social support	Model I + Reward	Model I + Job control, reward and social support
Contribution^a^ % (95% CI)	REF	−24 (−40;−6)	9 (−3; 20)	14 (−3; 24)	24 (9; 38)	23 (5; 40)
Mean differences (95% CI)						
Household income ($/year)						
≥100 000	REF	REF	REF	REF	REF	REF
60 000–99 999	0.32 (−0.10; 0.74)	0.61 (0.21; 1.01)**	0.28 (−0.13; 0.70)	0.25 (−0.16; 0.67)	0.19 (−0.22; 0.61)	0.22 (−0.20; 0.63)
40 000–59 999	0.62 (0.16; 1.07)**	0.93 (0.49; 1.37)**	0.56 (0.10; 1.03)*	0.55 (0.10; 1.01)*	0.32 (−0.13; 0.78)	0.39 (−0.07; 0.85)
0–39 999	1.26 (0.79; 1.73)***	1.56 (1.11; 2.02)***	1.15 (0.66; 1.63)***	1.09 (0.62; 1.55)***	0.95 (0.48; 1.42)***	0.96 (0.48; 1.45)***
Psychological demand (tertiles)						
0–7.2		REF				
7.3–10		0.92 (0.59; 1.26)***				
>10		2.44 (2.05; 2.83)***				
Job control (tertiles)						
≥25			REF			REF
21–24.9			−0.04 (−0.41; 0.32)			−0.20 (−0.57; 0.18)
0–20.9			0.46 (0.06; 0.85)*			0.00 (−0.42; 0.42)
Social support at work						
≥56				REF		REF
48–55				−0.01 (−0.38; 0.36)		−0.21 (−0.61; 0.19)
0–47				1.28 (0.89; 1.68)***		0.65 (0.19; 1.11)**
Working alone				0.35 (−0.48; 1.18)		−0.15 (−1.10; 0.79)
Reward (tertiles)						
>16					REF	REF
14–16					0.30 (−0.07; 0.66)	0.19 (−0.20; 0.58)
0–13					1.71 (0.34; 2.08)***	1.37 (0.94; 1.80)***

*p-value <0.05, **p-value <0.01, ***p-value <0.001

^a^Contribution calculated with this formula, (MD_basic_ – MD_adjusted_)/(MD_basic_), where MD_basic_ = Mean differences for age-adjusted models and MD_adjusted_ = Mean differences for models adjusted for work variables at each steps for the 0–39 999$ per year category of household income. Jackknife method was used to calculate 95% IC of the contributions

#### Other work-related factors

Table 4 presents the additional contributions of other psychosocial work-related factors and other work-related factors in the association between household income and psychological distress among men. Additional adjustments for other psychosocial work-related factors (Model VII) and for other work-related factors (Model VIII) do not increase the total contribution of JC, SS and reward in the association between household income and psychological distress (23% and 24% respectively).

**Table 4:** Contribution of other psychosocial work-related factors and other work-related factors to income inequalities in psychological distress among men

	Psychological distress	
	Model VII	Model VIII
	Model VI^a^ + Other psychosocial work-related factors^c^	Model VII + Other work-related factors^d^
Contribution^b^, % (95% CI)	23 (0; 45)	24 (−2; 47)
Mean differences (95% CI)		
Household income ($/year)		
≥100 000	REF	REF
60 000–99 999	0.21 (−0.18; 0.61)	0.21 (−0.19; 0.61)
40 000–59 999	0.56 (0.12; 1.00)*	0.55 (0.11; 1.00)*
0–39 999	0.97 (0.50; 1.43)***	0.96 (0.49; 1.44)***

*p-value <0.05, **p-value <0.01, ***p-value <0.001

^a^Model VI adjusted for age, job control, reward and social support (Table 3 and 4)

^b^Contribution calculated with this formula, (MD_basic_ – MD_adjusted_)/(MD_basic_), where MD_basic_ = Mean differences for age-adjusted models and MD_adjusted_ = Mean differences for models adjusted for work variables at each steps for the 0–39 999$ per year category for household income. Jackknife method was used to calculate 95% IC of the contributions

^c^Job contractual instability, psychological harassment, flexible schedule, paid leaves for sickness, emotionally demanding work, strain with public and possibility to do a work of quality

^d^Number of working hours, work schedule, noise exposure, solvent exposure and physical work constraints

## Discussion

The aims of the present study were to examine the contributions of psychosocial work factors from the DCS and the ERI models and of other work-related factors to social inequalities in psychological distress. The strongest social inequalities were observed in men, using household income as the SEP indicator. Psychosocial work factors from the DCS and the ERI models partly **e**xplained these inequalities. This contribution was higher in magnitude for reward, JC and SS. After considering psychosocial work factors from the DCS and ERI models, other psychosocial work-related factors and other work-related factors did not further contribute.

In the present study, social inequalities in psychological distress observed were of higher magnitude using household income. This is consistent with the findings of a meta-analysis which identified income as the socioeconomic indicator having the strongest inverse dose–response association with depression [5]. Income represents the flow of economic resources available to an individual [52], and persons with lower income are likely to have fewer resources for material needs [14]. Poor material living conditions may affect mental health through different mechanisms including poor social networks and a decreased access to health care services [53].

The findings of the present study indicate that psychosocial work factors are important contributors to SEP inequalities in psychological distress among men. In our study, income inequalities in psychological distress were attenuated after adjustment for reward, JC and SS, which is consistent with findings from previous studies [25, 27, 30, 34, 36]. (The results were similar with education inequalities, see Additional file 2).

The important contribution of reward found in the present study was in line with Niedhammer et al. who reported that reward contributed to explain 12.8% to 48.8% of social inequalities in depression among men [36]. However, in the present study, this component of the ERI model had the highest relative contribution. In a recent study in older workers, the contribution of ERI exposure was found to be higher in magnitude than that of job control, which is in line with our results [34]. While job control covers task-level characteristics, reward includes broader socioeconomic conditions, such as salaries, promotion prospects and job stability. It has been hypothesized that the adverse effects could be amplify when one feels that the ‘injustice’ is attributable to ‘out of control’ conditions [54]. Our findings suggest that insufficient reward at work could be an important pathway by which working in low-paid jobs leads to mental health problems. Studies with prospective design are needed to further test this hypothesis.

The contributions of the DCS dimensions, considered separately, were comparable to those reported in previous studies. JC was found to make the greatest contribution in explaining social inequalities in well-being and depression [30, 31, 36]. SS has also been shown to partly explain social inequalities in mental functioning [28, 36]. It is also noteworthy that previous studies have also observed an opposite effect of PD [15, 26, 28, 31, 36]. It suggests that high PD might not be particularly prevalent among workers with low SEP [28, 30, 31]. Consistent with this hypothesis, we found that PD was higher among people in the highest household income category (36% in the >100 000$/year category, compared to 20% in the 0–39 999$//year category), which could likely explain the inverse contribution found for PD.

In the current study, social inequalities in psychological distress were of smaller magnitude in women than in men. This finding is consistent with those of previous studies measuring SEP based on household income, occupation and/or education [55–57]. A potential explanation is that the relation between SEP and mental health for men and women differ depending on the SEP indicator used. While the SEP indicators used in the current study had little or no association with women’s mental health, other indicators such as the experience of current or childhood economic difficulties [58] and relative financial deprivation [59, 60] have been highlighted as important markers of mental health in women. Furthermore, other gender-related variables such as marital status and family responsibilities have previously been associated with SEP, working conditions and mental health. Indeed, among women, being a single parent has been associated with holding lower-grade hourly jobs [61] along with moderate to severe mental health disability [62]. Therefore, future research on social inequalities in women’s mental health may benefit from measuring a wider range of SEP indicators and stratifying or controlling for personal life factors (e.g., marital status, family responsibilities, and parity).

The addition of other work-related factors to the statistical models, including other psychosocial dimensions of the work environment, did not further explain social inequalities in psychological distress, over and above the contribution of DCS and ERI exposures. Previous studies have reported that work-related factors such as physical conditions [15, 31, 36] and working hours [29, 35] could contribute. However, in these previous studies, these factors were considered in separate models; therefore conclusions cannot be drawn on their independent effect. The present study suggests that adverse psychosocial work factors from the DCS and the ERI models might co-occur with those other deleterious exposures, partly explaining why they were shown to explain social inequalities in previous studies.

### Strengths and limitations of the study

The strengths of this study include: 1- It was conducted on a large sample of men and women working in a wide range of occupations, 2- the use of validated models to measure psychosocial work factors and psychological distress, 3- the use of three complementary SEP indicators, and 4- the evaluation of the contribution of the work environment on men and women health inequalities using an exhaustive set of work-related factors.

This study has some limitations. First, the study relies on a cross-sectional design. SEP and psychosocial work factors may contribute to the development of mental health, but workers with MHPs could also have limited access to more satisfying jobs and social status. Furthermore, since the study is cross-sectional, there is a potential for reverse causality, either by real changes in the work environment or by changes in the evaluation of the same work environment for individuals with MHP [63] However, it should be noted that available prospective studies strongly support the temporal precedence of adverse psychosocial work exposures, prior to the onset of MHP [10–13]. Second, information on some MHP risk factors, such as personal and family history of MHP, was not available possibly leading to residual confounding. Third, the exposure and outcome were self-reported, possibly introducing a common method bias [64] leading to inflated measures of effect. However, using measures of psychological distress rather than the use of a formal diagnosis or other measures of a diagnosed mental health problem is very pertinent for screening and prevention of mental health problems before the onset of more severe form of diseases. Fourth, the participation was suboptimal. We can therefore not exclude the possibility of selection bias. Estimates were weighted to maintain the representativeness of the Quebec workforce in terms of socioeconomic distribution, minimizing this possibility [64]. Lastly, workers exposed to adverse working conditions as well as prevalent cases of MHP might have switched to less exposed jobs or left employment. This healthy worker survivor effect could have led to an underestimation of the contribution of psychosocial work factors and other work characteristics to mental health inequalities [65].

## Conclusion

In this population-based study, low household income and low education were associated with high psychological distress among men. Psychosocial work factors from the DCS and the ERI models contributed to explain these social inequalities in mental health. Psychosocial factors at work are frequent and modifiable. The present study supports the relevance of targeting these factors for the primary prevention of MHP and for health policies aiming to reduce social inequalities in mental health.

## Additional files

Additional file 1: Work-related variables included in the analyses. (DOCX 27 kb)
Additional file 2: Contribution of work factors in the education inequalities in psychological distress among men. (DOCX 22 kb)

## Abbreviations

CI: Confidence interval
DCS: Demand-Control-Support
ERI: Effort-Reward Imbalance
JC: Job control
JCQ: Job content questionnaire
MD: M﻿ean differences
MHP: Mental health problems
PD: Psychological demands
SEP: Socioeconomic position
SS: Social support
