# Supplementary material for: Psychosocial work factors and social inequalities in psychological distress: a population-based study
Source: BMC Public Health. 2017 Jan 18;17:91. doi: 10.1186/s12889-017-4014-4 (PMC5241997; doi:10.1186/s12889-017-4014-4)
Supplement: Additional file 1: — Work-related variables included in the analyses. (DOCX 27 kb) [file 12889_2017_4014_MOESM1_ESM.docx]

**Additional file 1.** Work-related variables included in the analyses

| **Work-related factor** | **Items** | **Response choices** |
| --- | --- | --- |
| **Psychological demand** | Items from the short version of the JCQ:  - “Work fast”  - “Excessive work”  - “Enough time”  - “Conflicting demands”  - “Work hard”  Item from the long version of the JCQ:  - “Tasks interrupted” | 1-strongly disagree  2-disagree  3-agree  4- strongly agree |
| **Job control** | Skill discretion:  - “Learn new things”  - “High skill level”  - “Repetitive work”  Decision authority:  - “Allows own decisions”  - “A lot of say” | 1-strongly disagree  2-disagree  3-agree  4- strongly agree |
| **Social support** | Co-worker social support:  - “Co-workers helpful”  - “Hostile co-workers”  Supervisor social support:  - “Supervisor good organizer”  - “Supervisor pays attention”  - “Helpful supervisor”  - “Hostile supervisor”  Item from the COPSOC :  - Are you part of a group in your work? | 1-strongly disagree  2-disagree  3-agree  4- strongly agree |
| **Reward** | - I receive the respect I deserve from my superior or a respective relevant person.  - My job promotion prospects are poor.  - My employment security is poor  - Considering all my efforts and achievements, I receive the respect and prestige I deserve at work.  - Considering all my efforts and achievements, my job promotion prospects are adequate  - Considering all my efforts and achievements, my salary / income is adequate.  Items of COPSOC:  - At work, my efforts are sufficiently appreciated  - At work, I am treated equitably | 1-strongly disagree  2-disagree  3-agree  4- strongly agree |
| **Job contractual instability** | - Working part-time (15 to 29 hours per week) and wanting more work hours  - Obtained job by an agency  - Has a fixed term employment | Yes: if the response was yes to at least one of the three items  No: if the response was no to all the three items |
| **Psychological harassment** | During the past 12 months at your current main employment, were you subjected to psychological harassment, that is, repeated verbal harassment or actions that affected your dignity or personal integrity? | Yes  No |
| **Flexible work schedule** | Are you aware if the following are available to you in your workplace?  - Flexible work schedule | Yes  No |
| **Paid leave for sickness** | Are you aware if the following are available to you in your workplace?  - Medical leave paid by employer | Yes  No |
| **Emotionally demanding work** | I find my work emotionally demanding | 1-strongly disagree  2-disagree  3-agree  4- strongly agree |
| **Strain with public** | How often do you experience tense situations in your relations with the public? | 1-never  2-occasionally  3-often  4-always |
| **Possibility to do a work of quality** | In your current main employment, would you say that you have the means to do quality work? | 1-never  2-occasionally  3-often  4-always |
| **Work schedule** | Which of the following statements best describes your work schedule? | 1- Regular schedule on day only  2- Regular schedule with evening work  3- Regular schedule with night work  4- Rotating schedule  5- Other schedule |
| **Number of working hours** | On average, how many hours per week do you usually work for all your jobs? | 1- <30 h  2- 30-39 h  3- 40 h  4- >40 h |
| **Self-reported exposure to noise** | In your main employment, how often are you working in an environment where it is so noisy that it is difficult to hold a conversation with someone a few feet or one meter from you, even when shouting? | 1-never  2-occasionally  3-often  4-always |
| **Self-reported exposure to solvents** | In your main employment, how often are you inhaling vapors of solvents such as paint strippers, oil paint, thinners, varnish, Varsol, turpentine, etc.? | 1-never  2-occasionally  3-often  4-always |
| **Physical constraints** | In the context of your main employment, how often are you exposed to each of the following situations  - Work with your hands over your shoulders  - Work with your back bent  - Perform repetitive movements  - Perform movements that require precision  - Make a physical effort  - Handle loads without assistance  - Vibrations from hand tools  - Whole body vibration  - Work posture and opportunity to sit | 1-never  2-occasionally  3-often  4-always |
