# Supplementary material for: Psychosocial work factors and social inequalities in psychological distress: a population-based study
Source: BMC Public Health. 2017 Jan 18;17:91. doi: 10.1186/s12889-017-4014-4 (PMC5241997; doi:10.1186/s12889-017-4014-4)
Supplement: Additional file 2: — Contribution of work factors in the education inequalities in psychological distress among men. (DOCX 22 kb) [file 12889_2017_4014_MOESM2_ESM.docx]

**Additional file 2.** Contribution of work factors in the education inequalities in psychological distress among men

**Table 4A.** Contribution of the Demand-Control-Support and the Effort-Reward Imbalance models to education inequalities in psychological distress among men.

|  | **Model I** | **Model II** | **Model III** | **Model IV** | **Model V** | **Model VI** |
| --- | --- | --- | --- | --- | --- | --- |
|  | Age-adjusted | Model I + Psychological demand | Model I + Job control | Model I + Social support | Model I + Reward | Model I + Job control, reward and social support |
| **Contribution^1^, % (95% CI)** | REF | -29 (-62; 23) | 39 (-20; 78) | 33 (-16; 66) | 19 (-13; 43) | 34 (-23; 74) |
| **Prevalence ratio, PR (95% CI)** |  |  |  |  |  |  |
| Education degree |  |  |  |  |  |  |
| University  College  High school degree  Less than high school degree | REF  0.00 (-0.42; 0.43)  0.21 (-0.18; 0.61)  0.56 (0.06; 1.05)* | REF  0.07 (-0.34; 0.47)  0.33 (-0.05; 0.72)  0.72 (0.24; 1.19)** | REF  -0.05 (-0.47; 0.37)  0.08 (-0.33; 0.48)  0.34 (-0.17-0.85) | REF  -0.05 (-0.47; 0.37)  0.10 (-0.29; 0.50)  0.37 (-0.12; 0.86) | REF  -0.12 (-0.53; 0.30)  0.13 (-0.26; 0.52)  0.45 (-0.03; 0.93) | REF  -0.12 (-0.54; 0.29)  0.11 (-0.29; 0.51)  0.37 (-0.13; 0.87) |
| Psychological demand (tertiles)  0-7.2  7.3-10  >10 |  | REF  0.94 (0.60; 1.27)***  2.29 (1.91; 2.68)*** |  |  |  |  |
| Job control (tertiles)  ≥25  21-24.9  0-20.9 |  |  | REF  0.12 (-0.24; 0.48)  0.73 (0.34; 1.12)*** |  |  | REF  -0.08 (-0.45; 0.29)  0.17 (-0.24; 0.57) |
| Social support at work  ≥56  48-55  0-47  Working alone |  |  |  | REF  0.00 (-0.36; 0.37)  1.34 (0.95; 1.73)***  0.25 (-0.56; 1.05) |  | REF  -0.29 (-0.67; 0.10)  0.57 (0.12-1.02)*  -0.25 (-1.16-0.65) |
| Reward (tertiles)  >16  14-16  0-13 |  |  |  |  | REF  0.35 (0.00; 0.71)*  1.85 (1.49; 2.21)*** | REF  0.25 (-0.13; 0.63)  1.50 (1.08; 1.92)*** |

^1^ Contribution calculated with this formula: (MD_basic_ – MD_adjusted_)/(MD_basic_ ), where MD_basic_ = Mean differences for age-adjusted models and MD_adjusted_ = Mean differences for models adjusted for work variables at each steps for the 0-39 999$ per year category of household income. Jackknife method was used to calculate 95% IC of the % contribution.

*p-value <0.05, **p-value <0.01, ***p-value <0.001

**Table 5A**. Contribution of other psychosocial work-related factors and other work-related factors to in education inequalities in psychological distress among men

|  | **Psychological distress** | |
| --- | --- | --- |
|  | **Model VII** | **Model VIII** |
|  | Model VI^1^ +  **Other psychosocial work-related factors**^3^ | Model VII +  **Other work-related factors**^4^ |
| **Contribution^2^, % (95% CI)** | 26 (-39; 80) | 64 (-33; 131) |
| **Education degree, MD (95%CI)**  University  College  High school degree  Less than high school degree | REF  -0.08 (-0.47; 0.32)  0.16 (-0.23; 0.56)  0.41 (-0.08; 0.90) | REF  -0.15 (-0.56; 0.26)  -0.01 (-0.44; 0.42)  0.20 (-0.32; 0.72) |

^1^ Model VI adjusted for age, job control, reward and social support (Table 3 and 4)

^2^ Contribution calculated with this formula: (MD_basic_ – MD_adjusted_)/(MD_basic_ ), where MD_basic_ = Mean differences for age-adjusted models and MD_adjusted_ = Mean differences for models adjusted for work variables at each steps for the 0-39 999$ per year category of household income. Jackknife method was used to calculate 95% IC of the % contribution.

^3^ Job contractual instability, psychological harassment, flexible schedule, paid leaves for sickness, emotionally demanding work, strain with public and possibility to do a work of quality

^4^ Number of working hours, work schedule, noise exposure, solvent exposure and physical work constraints

*p-value <0.05, **p-value <0.01, ***p-value <0.001
